# Impact of concomitant fibrates on immunotherapy outcomes for advanced non‐small cell lung cancer

**DOI:** 10.1002/cam4.4847

**Published:** 2022-05-24

**Authors:** William A. Stokes, Madhusmita Behera, Renjian Jiang, David A. Gutman, Zhonglu Huang, Abigail Burns, Nikhil T. Sebastian, Vidula Sukhatme, Michael C. Lowe, Suresh S. Ramalingam, Vikas P. Sukhatme, Drew Moghanaki

**Affiliations:** ^1^ Department of Radiation Oncology Emory University Atlanta Georgia USA; ^2^ Winship Cancer Institute Emory University Atlanta Georgia USA; ^3^ Atlanta Veterans Affairs Health Care System Decatur Georgia USA; ^4^ Morningside Center for Innovative and Affordable Medicine Emory University Atlanta Georgia USA; ^5^ GlobalCures, Inc Newton Massachusetts USA; ^6^ Division of Surgical Oncology Emory University Atlanta Georgia USA; ^7^ Department of Hematology and Medical Oncology Emory University Atlanta Georgia USA; ^8^ Department of Radiation Oncology University of California Los Angeles Los Angeles California USA

**Keywords:** fibrates, immune checkpoint inhibitors, peroxisome proliferator‐activated receptor (PPAR) agonists

## Abstract

**Background:**

Peroxisome proliferator‐activated receptor agonists such as fibrates restore oxidative metabolism in cytotoxic T‐lymphocytes, thereby enhancing response to immune checkpoint inhibitors (ICI) in preclinical models. However, there is no evidence in humans on the clinical impact of fibrates as an adjunct to ICI.

**Methods:**

In this cohort study of Veterans with non‐small cell lung cancer (NSCLC) receiving ICI, fibrate exposure was defined as a prescription filled within 90 days of an ICI infusion. Overall survival (OS), measured from the start of ICI, was compared between exposed and unexposed Veterans. Cox multivariable analysis (MVA) was used to identify factors associated with OS. A sensitivity analysis of Veterans with stage IV NSCLC who received docetaxel without ICI was similarly performed.

**Results:**

The ICI cohort included 3593 Veterans, of whom 301 (8.5%) coincidentally received a fibrate. Veterans receiving fibrates were more likely to be older, white, male, and married, and to have greater comorbidity burden, but less likely to receive chemotherapy. Coincidental fibrates were associated with improved OS both on MVA (HR 0.86, 95%CI 0.75–0.99) and in a matched subset (HR 0.75, 95%CI 0.63–0.90). In contrast, among the cohort of 968 Veterans treated with chemotherapy, fibrates did not have a significant impact on OS by MVA (HR 0.99, 95%CI 0.79–1.25) or in a matched subset (HR 1.02, 95%CI CI 0.75–1.39).

**Conclusions:**

Concomitant fibrates are associated with improved OS among NSCLC patients receiving ICI but not among those receiving chemotherapy. This hypothesis‐generating observation supports a potential role for fibrates as an adjunct to immunotherapy.

## Graphical abstract

Fibrates concomitant to immune checkpoint inhibitors are associated with improved survival in patients with non‐small cell lung cancer.

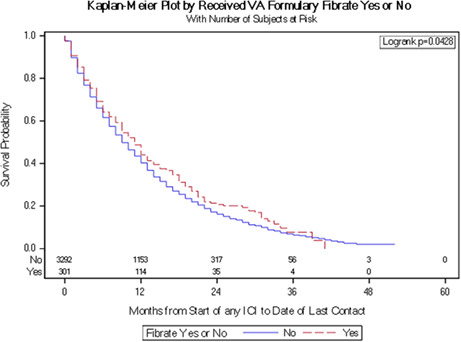

## 1 INTRODUCTION

The past decade has witnessed a dramatic expansion in the armamentarium of drugs available to treat non‐small cell lung cancer (NSCLC), translating to measurable improvements in mortality at the population level.^1^ For the majority of patients with advanced NSCLC whose tumors lack an actionable driver alteration, immune checkpoint inhibitors (ICI) now constitute the mainstay of first‐line therapy.^2^ However, durable benefit with ICI therapy is limited to a minority of patients: The five‐year survival rate for patients with high programmed death ligand‐1 (PD‐L1) expression is 32% with an objective response, and disease progression happens in a majority.^3^, ^4^, ^5^, ^6^ Ultimately, despite the tremendous therapeutic advances over the past decade, there remains an unmet need for strategies to increase the number and duration of responses to ICI. A major thrust of ongoing research efforts involves the development of novel combination approaches to improve the efficacy of ICI.

Preclinical research has highlighted cytotoxic T‐lymphocyte metabolism as a potential therapeutic target. Oxidative phosphorylation is critical for the energy‐intensive effector and memory functionality of T‐lymphocytes. However, exhausted T‐lymphocytes contain mitochondria that exhibit insufficient oxidative metabolism, which can only be partially and temporarily reversed by PD‐1 blockade.^7^ Induction of peroxisome‐proliferator activated receptor (PPAR)‐γ coactivator 1α (PGC1α) in these lymphocytes reprograms mitochondrial metabolism, leading to restoration of oxidative metabolism and antitumor efficacy.^7^ Fibrate drugs, which activate PPARα and in turn PGC1α, enhance the efficacy of PD‐1 blockade in mouse models, leading to suppression of tumor growth and increase in host survival.^8^, ^9^ Mechanistically, this effect seems mediated by enhanced oxidative metabolism by mitochondria, leading to increased proliferation and survival of effector T‐lymphocytes.^9^

Fibrates, therefore, constitute a novel potential therapeutic strategy to enhance response to immunotherapy. However, there is currently no evidence in human populations on their clinical impact as an adjunctive strategy with ICI. We, therefore, aimed to evaluate the impact of coincidental fibrate use in NSCLC patients receiving ICI using real‐world evidence from clinical records available in the national Veterans Health Administration (VHA) database.

## 2 METHODS

The VHA is the largest integrated health care system in the United States (US) and serves nine million Veterans annually, approximately 50,000 with cancer. Clinical and administrative data are routinely collected on each VHA enrollee from over 150 VHA hospitals and thousands of clinics into the Corporate Data Warehouse (CDW). We queried this resource for NSCLC diagnoses occurring from 2010, to capture patients with metachronous presentation of advanced/metastatic NSCLC, through 2018, to allow for sufficient follow‐up. This study was conducted with the approval of the local institutional review board prior to data acquisition and analysis.

We defined a nested cohort of patients with NSCLC who received ICI. Utilization of ICI was ascertained from the CDW using the secure VA Informatics and Computing Infrastructure workspace, focusing on the four agents approved for use in NSCLC through 2018 (nivolumab, pembrolizumab, durvalumab, atezolizumab). Exposure to fibrates was defined as the Veteran having an active prescription for a fibrate at any time within the window from 90 days before to 90 days after any ICI infusion. To mitigate the impact of immortal time bias, patients whose initial fibrate prescription occurred more than 60 days after the start of ICI were excluded. Patients were categorized *a priori* into groups for sociodemographic factors (age, race, gender, population density, employment status, marital status), clinical characteristics (Elixhauser comorbidity index^10^ with relevant comorbidities ascertained from the CDW), cancer‐specific features (histology, stage at diagnosis, year of diagnosis), and treatment‐related variables (time from diagnosis to ICI initiation, sequence of ICI with respect to chemotherapy). Pearson's χ^2^ tests were used to assess associations between variables and fibrate exposure.

For comparison, we conducted a parallel nested cohort study of NSCLC patients who were diagnosed with stage IV disease and received docetaxel‐based chemotherapy but not ICI. Docetaxel utilization was ascertained in the same manner as ICI in the first cohort. Fibrate exposure was defined with respect to the initiation of docetaxel and compared according to covariates as above.

Within each cohort, the primary outcome was overall survival, measured from the date of initial ICI or docetaxel administration to the date of last follow‐up or death. Vital status was ascertained from Department of Defense data. Median follow‐up time was estimated according to the reverse Kaplan–Meier method. Survival was first estimated using the Kaplan–Meier method without adjustment or matching for covariates and was compared between groups using log‐rank test. Univariable Cox proportional hazards regression was then performed, with survival associations expressed as hazard ratios (HR) and corresponding 95% confidence intervals (95%CI), and HR > 1 indicating greater risk of mortality. Using backward selection with an α level of removal of 0.05, multivariable Cox proportional hazards regression was then performed. Statistical analyses were performed using SAS Enterprise Guide 7.1 (SAS Institute Inc.). Tests were two‐sided with a level of significance of *p* = 0.050.

Generalized propensity scores were separately calculated for the ICI and docetaxel cohorts by multinomial logistic regression, treating fibrate exposure as outcome and covariates as predictors. A generalized propensity score matching (PSM) algorithm was applied to create a pseudo‐sample where the aforementioned sociodemographic, clinical, cancer‐specific, and treatment‐related covariates were balanced among the comparison groups. The covariate balance was checked before and after PSM by the standardized difference, with values <0.2 considered an acceptable imbalance. Associations with survival were then examined in the matched samples using Kaplan–Meier and Cox proportional hazards regression as above.

## 3 RESULTS

### 3.1 ICI Cohort

In total, 3593 Veterans with NSCLC receiving ICI were identified, of whom 301 (8.5%) received concurrent fibrate. The median follow‐up time from the start of ICI was 8 months. A majority of the cohort was older than 65 (73.7%), white (72.8%), and male (97.0%), lived in urban areas (66.1%), was diagnosed with NSCLC after 2015 (57.6%), and had received chemotherapy prior to ICI (53.8%). A plurality of the cohort was not employed (42.2%), had an Elixhauser comorbidity index of ≤4 (28.3%), had adenocarcinoma histology (47.6%), and had stage IV disease at diagnosis (40.9%). Nivolumab (59.5%) and pembrolizumab (35.0%) were the most common ICI agents, followed by durvalumab (6.8%) and atezolizumab (3.3%). Among the 301 patients with fibrate exposure, gemfibrozil was the most common agent (95.7% of fibrate cohort), followed by fenofibrate (3.3%) and multiple fibrates (1.0%). There were significant baseline differences between fibrate‐exposed and ‐unexposed patients, with exposed patients significantly more likely to be older, white, male, and married; to have higher comorbidity burden; and to have started ICI within 4 months of NSCLC diagnosis; they were significantly less likely to have received chemotherapy before ICI (all *p* ≤ 0.012; Table 1).

**TABLE 1:** Descriptive statistics of unmatched cohort receiving immune checkpoint inhibitors, stratified by fibrate exposure (χ^2^)

		Fibrate				
Variable	Categories	No *N* = 3292		Yes *N* = 301		
		*N*	%	*N*	%	*p*
Age	≤65	895	27.2	49	16.3	<0.001
	66–70	1024	31.1	97	32.2	
	71–75	780	23.7	93	30.9	
	>75	593	18.0	62	20.6	
Race	White	2372	72.1	244	81.1	0.008
	Black	700	21.3	41	13.6	
	Other	46	1.4	4	4.3	
	Unknown	174	5.3	12	4.0	
Gender	Male	3185	96.7	299	99.3	0.012
	Female	107	3.3	2	0.7	
Geography	Urban	2185	66.4	190	63.1	0.254
	Rural	1107	33.6	111	36.8	
Employment	Employed	683	20.7	50	16.6	0.314
	Not employed	1383	42.0	132	43.9	
	Retired	1118	34.0	111	36.9	
	Unknown	108	3.3	8	2.7	
Marital status	Married	1535	46.6	154	51.2	0.003
	Not married	1755	53.3	145	48.2	
	Unknown	2	0.1	2	0.7	
Elixhauser comorbidity index	0–4	970	29.5	47	15.6	<0.001
	5–6	732	22.2	68	22.6	
	7–9	854	25.9	83	27.6	
	>9	736	22.4	103	34.2	
Histology	Squamous cell carcinoma	1219	37.0	95	31.6	0.168
	Adenocarcinoma	1558	47.3	154	51.2	
	Other	515	15.6	52	17.3	
Stage at diagnosis	0	4	0.1	0	0.0	0.162
	I	406	12.3	44	14.6	
	II	243	7.4	17	5.6	
	III	890	27.0	82	27.2	
	IV	1358	41.3	110	36.5	
	Unknown	391	11.9	48	16.0	
Year of diagnosis	2010–2015	1400	42.5	124	41.2	0.655
	2016–2018	1892	57.5	177	58.8	
Months from diagnosis to ICI	0–4	766	23.3	94	31.2	0.004
	5–10	956	29.0	64	21.3	
	11–19	716	21.7	62	20.6	
	>19	854	25.9	81	26.9	
Chemotherapy	None	418	12.7	56	18.6	0.006
	Before ICI	1796	54.6	138	45.8	
	During ICI	995	30.2	101	33.6	
	After ICI	83	2.5	6	2.0	

Abbreviation: ICI, immune checkpoint inhibitor.

**FIGURE 1:**
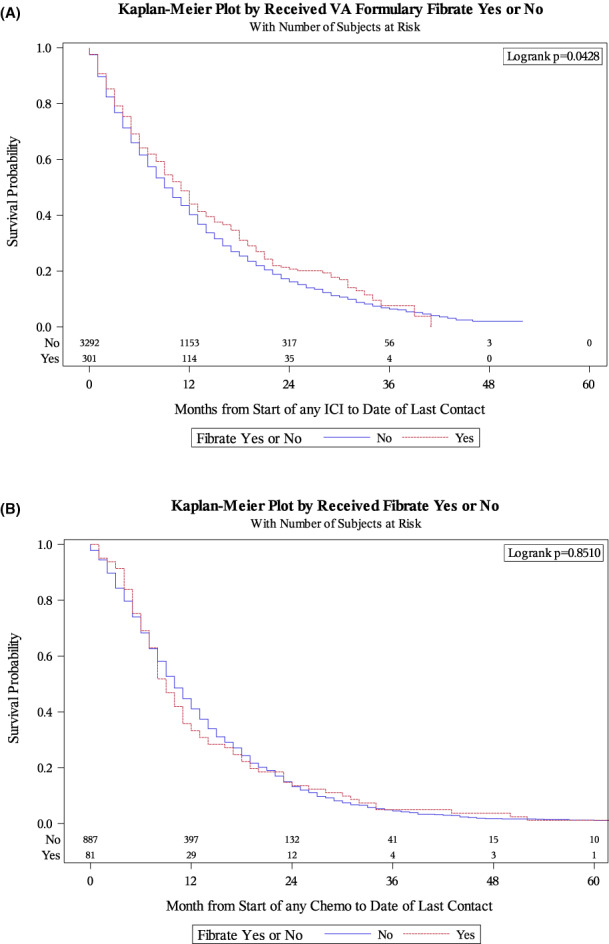
Overall survival by fibrate exposure in patients receiving (A) immune checkpoint inhibitors and (B) docetaxel

Median OS was significantly longer among Veterans receiving fibrates at 11 versus 9 months (log‐rank *p* = 0.043) (Figure 1A). On Cox univariable analysis fibrate exposure was associated with improved OS; however, the association did not attain the level of statistical significance (HR 0.87, 95%CI 0.76–1.00, *p* = 0.051; Table 2). Factors significantly associated with longer OS were older age, black race, lower comorbidity burden, adenocarcinoma histology, earlier stage at diagnosis, earlier year of diagnosis, longer time from diagnosis to ICI, and receipt of chemotherapy during or after ICI.

**TABLE 2:** Cox regression analysis of overall survival for unmatched patients treated with immune checkpoint inhibitors

Variable	Categories		UVA			MVA			
		*N*	HR	95%CI	*p*	HR	95%CI	*p*	pT3
Fibrate	No	3292	—	—	—	—	—	—	0.042
	Yes	301	0.87	0.76–1.00	0.051	0.86	0.75–0.99	0.042	
Age	≤65	944	1.20	1.07–1.35	0.001	1.40	1.24–1.59	<0.001	<0.001
	66–70	1121	1.22	1.09–1.36	<0.001	1.31	1.17–1.47	<0.001	
	71–75	873	0.88	0.78–0.99	0.032	0.91	0.81–1.03	0.139	
	>75	655	—	—	—	—	—	—	
Race	White	2616	—	—	—	—	—	—	0.004
	Black	741	0.86	0.78–0.95	0.002	0.85	0.77–0.93	<0.001	
	Other	50	1.01	0.73–1.40	0.959	0.94	0.68–1.31	0.721	
	Unknown	186	1.05	0.89–1.24	0.578	1.09	0.92–1.29	0.302	
Gender	Male	3484	—	—	—	—	—	—	0.011
	Female	109	0.84	0.67–1.06	0.148	0.73	0.58–0.93	0.011	
Geography	Urban	2375	—	—	—				
	Rural	1218	1.05	0.97–1.13	0.266				
Employment	Employed	733	—	—	—	—	—	—	0.004
	Not employed	1515	1.08	0.97–1.19	0.163	1.05	0.94–1.16	0.381	
	Retired	1229	1.11	1.00–1.24	0.047	1.21	1.08–1.35	<0.001	
	Unknown	116	1.17	0.94–1.47	0.167	1.12	0.89–1.40	0.345	
Marital status	Married	1689	—	—	—				
	Not married	1900	0.99	0.92–1.06	0.722				
	Unknown	4	1.18	1.44–3.15	0.739				
Elixhauser comorbidity index	0–4	1017	—	—	—	—	—	—	<0.001
	5–6	800	1.12	1.01–1.25	0.034	1.15	1.03–1.28	0.012	
	7–9	937	1.34	1.21–1.49	<0.001	1.31	1.18–1.45	<0.001	
	>9	839	1.23	1.11–1.37	<0.001	1.23	1.10–1.36	<0.001	
Histology	Squamous cell carcinoma	1314	—	—	—	—	—	—	<0.001
	Adenocarcinoma	1712	0.83	0.77–0.90	<0.001	0.82	0.75–0.89	<0.001	
	Other	567	0.96	0.86–1.07	0.437	0.92	0.82–1.09	0.134	
Stage at diagnosis	0	4	0.42	0.11–1.69	0.223	0.49	0.12–1.96	0.312	<0.001
	I	450	0.89	0.78–1.00	0.048	0.99	0.87–1.13	0.896	
	II	260	0.88	0.75–1.02	0.090	0.94	0.80–1.10	0.455	
	III	972	0.83	0.76–0.91	<0.001	0.83	0.75–0.92	<0.001	
	IV	1468	—	—	—	—	—	—	
	Unknown	439	1.08	0.95–1.22	0.224	1.13	1.00–1.28	0.051	
Year of diagnosis	2010–2015	1524	0.81	0.75–0.87	<0.001	0.87	0.79–0.96	0.006	0.006
	2016–2018	2069	—	—	—	—	—	—	
Months from diagnosis to ICI	0–4	860	1.39	1.24–1.54	<0.001	1.28	1.10–1.48	0.001	0.014
	5–10	1020	1.30	1.18–1.44	<0.001	1.15	1.02–1.31	0.026	
	11–19	778	1.16	1.05–1.30	0.005	1.10	0.98–1.24	0.102	
	>19	935	—	—	—	—	—	—	
Chemotherapy	None	474	1.06	0.94–1.19	0.337	0.95	0.84–1.08	0.422	<0.001
	Before ICI	1934	—	—	—	—	—	—	
	During ICI	1096	0.73	0.67–0.79	<0.001	0.71	0.65–0.77	<0.001	
	After ICI	89	0.60	0.47–0.78	<0.001	0.55	0.43–0.72	<0.001	

Abbreviations: 95%CI, 95% confidence interval; HR, hazard ratio; ICI, immune checkpoint inhibitor; MVA, multivariable analysis; pT3, type‐3 *p*‐value; UVA, univariable analysis.

On Cox multivariable analysis, fibrate receipt was significantly associated with improved OS (HR 0.86, 95%CI 0.75–0.99, *p* = 0.042; Table 2). Other factors correlated to longer OS were older age, black race, female gender, employed status, lower comorbidity burden, adenocarcinoma histology, earlier stage and year of diagnosis, greater time from diagnosis to ICI, and receipt of chemotherapy during or after ICI.

**FIGURE 2:**
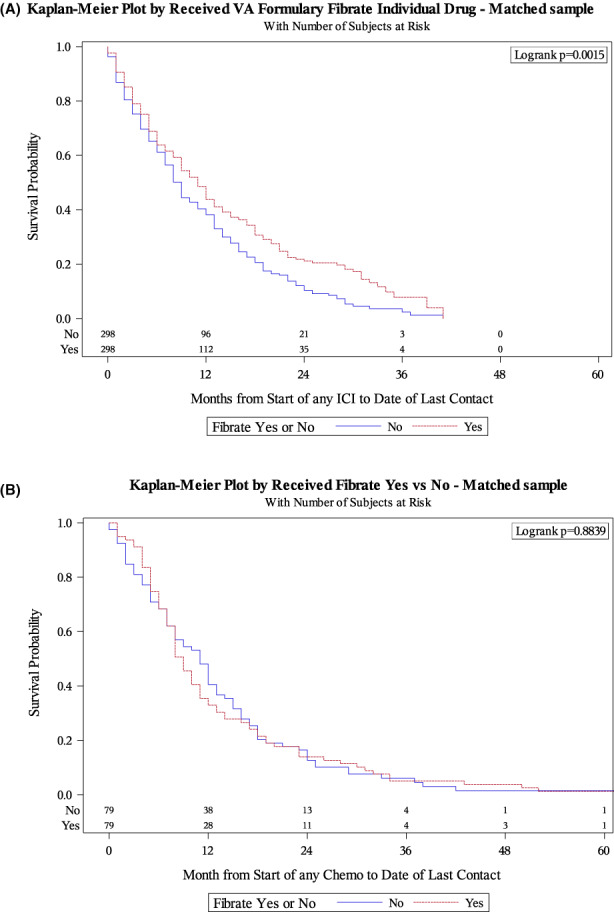
Overall survival by fibrate exposure in propensity‐matched cohorts of patients receiving (A) immune checkpoint inhibitors and (B) docetaxel

Propensity‐score matching yielded 298 fibrate‐exposed Veterans well‐matched to 298 unexposed individuals (Table S1). In this matched subset, fibrate‐exposed patients experienced significantly longer OS than their unexposed counterparts at a median of 11 versus 9 months (log‐rank *p* = 0.002) (Figure 2A). Likewise, Cox regression for this subset demonstrated improved OS among patients taking fibrate (HR 0.75, 95%CI 0.63–0.90, *p* = 0.001; Table 3).

**TABLE 3:** Cox regression analyses of overall survival for propensity‐matched cohorts treated with immune checkpoint inhibitors or docetaxel

		ICI cohort				Docetaxel cohort			
Variable	Categories	UVA				UVA			
		*N*	HR	95%CI	*p*	*N*	HR	95%CI	*p*
Fibrate	No	298	—	—	—	79	—	—	—
	Yes	298	0.75	0.63–0.90	0.001	79	1.02	0.75–1.39	0.885

Abbreviations: 95%CI, 95% confidence interval; HR, hazard ratio; ICI, immune checkpoint inhibitor; UVA, univariable analysis.

### 3.2 Docetaxel cohort

A total of 968 Veterans were identified who were diagnosed with stage IV NSCLC and treated with docetaxel but not ICI, of whom 81 (8.4%) received fibrates. Median follow‐up from start of docetaxel was 10 months. A majority of this cohort was older than 65, white, and male, lived in urban areas, were not married, had adenocarcinoma histology, and were diagnosed with NSCLC before 2015, while a plurality was not employed and had an Elixhauser comorbidity index of 4 or 5. Among the 81 patients with fibrate exposure, gemfibrozil was the most common agent (80.2% of fibrate cohort), followed by fenofibrate (13.6%) and multiple fibrates (6.2%). There were significant baseline differences between fibrate‐exposed and unexposed patients, with exposed patients significantly more likely to be white and to have higher comorbidity burden (both *p* ≤ 0.040; Table S2).

Median OS in the docetaxel cohort did not significantly differ between Veterans receiving versus not receiving fibrates at 9 versus 10 months (log‐rank *p* = 0.851) (Figure 1B). Similarly, on Cox univariable analysis, fibrate exposure was not associated with OS (HR 1.02, 95%CI 0.81–1.28, *p* = 0.855; Table S3). Factors significantly associated with OS included time from fibrate to chemotherapy, age, gender, histology, and year of diagnosis.

On Cox multivariable analysis, fibrate exposure was not associated with OS (HR 0.99, 95%CI 0.79–1.25 *p* = 0.962; Table S3). Factors correlated with longer OS were adenocarcinoma histology and earlier year of diagnosis.

Propensity‐score matching yielded 79 fibrate‐exposed Veterans well‐matched to 79 unexposed individuals (Table S4). In this matched subset, OS did not significantly differ between fibrate‐exposed Veterans and unexposed Veterans at a median of 9 versus 11 months (log‐rank *p* = 0.884) (Figure 2B). Fibrate exposure was also not associated with OS on Cox regression (HR 1.02, 95%CI 0.75–1.39, *p* = 0.885; Table 3).

## 4 DISCUSSION

In the first study of its kind, we demonstrate a modest increase in OS for patients who received ICI with concomitant fibrates, an association that was not observed when fibrates were given concomitant with chemotherapy. This real‐world evidence supports the hypothesis and pre‐clinical observations that PPAR agonists induce reprogramming of mitochondrial metabolism in exhausted cytotoxic T‐lymphocytes to restore their antitumor activity in patients treated with ICI. Our observations raise the intriguing possibility of using fibrates or other PPAR agonists as an adjunctive strategy to enhance response to ICI.

The association of fibrates with the improved outcomes when prescribed concurrent to ICI, but not to chemotherapy, suggests that the effect of fibrates is specific to patients receiving immunotherapy and not related to a generalized antitumor effect or other clinical impacts that could confound the observation of improved OS. We are not aware of any evidence indicating a direct anticancer effect of fibrates in patients with NSCLC. A growing body of preclinical evidence indicates that fibrates exert an immunologic effect by modulating T‐lymphocyte metabolism. At baseline, the hypoxic nature of the tumor microenvironment inhibits oxidative phosphorylation by mitochondria. Whereas tumor cells can exploit glycolysis to continue to meet their energetic needs, T‐lymphocytes depend on mitochondrial respiration and are functionally impaired by hypoxia.^11^ Anti‐PD‐1 monotherapy delays tumor progression but does not alter T‐lymphocyte metabolism. However, exposing T‐lymphocytes to PPAR agonists such as fibrates restores oxidative phosphorylation and enhances the efficacy of anti‐PD‐1 treatment.^7^, ^11^ Our clinical observations in humans are concordant with an immunologic effect for fibrates.

In comparison to newly developed agents, fibrates offer the advantages of prior approval by regulatory bodies, established and tolerable toxicity profiles,^12^ widespread availability,^13^ oral administration route, off‐patent status, and low cost. Cost in particular has recently been identified as a burgeoning threat to the field of oncology, with a mean price for a single course of therapy for one patient exceeding $150,000.^14^ The field's reliance on novel agents to advance innovation in oncology has led some to forecast dire fiscal consequences and the need for radical changes to payment systems.^14^, ^15^ In contrast, repurposing existing drugs such as fibrates for cancer treatment holds the promise of enhancing outcomes at a mere fraction of the cost.^16^ Even weighed against a potentially smaller clinical benefit than we observe in our study, these advantages would translate to a favorable cost–benefit ratio at both patient and system levels.

Our study's primary strength lies in its population drawn from a national integrated health system in which covered patients receive a majority of their care, including prescription medications. These features allow robust capture of both medication and follow‐up data. Yet several key limitations deserve emphasis.

The primary limitation of our analysis lies in its retrospective cohort design, which renders it susceptible to selection bias. Notably, fibrates are generally recommended only for patients with severe hypertriglyceridemia refractory to other treatment approaches such as statins,^17^, ^18^ and fibrate exposure may accordingly indicate adverse cardiometabolic risk. Our finding of improved outcomes among fibrate‐exposed patients despite this risk is therefore noteworthy and suggests that the oncologic benefit of fibrates delivered concurrently to ICI outweighs any negative impact from cardiovascular risk. Importantly, these conflicting impacts on OS may account for the relatively small effect size we observed and raise the possibility that the true benefit of fibrates may be larger than we are able to determine from this retrospective study.

Second, unmeasured confounders may have biased our results. We were not able to ascertain tumor PD‐L1 expression or tobacco abuse from the CDW. The reduced receipt of chemotherapy with ICI among our fibrate cohort may reflect higher PD‐L1 expression, which would portend better response to ICI. Alternatively, the older age and greater comorbidity burden of fibrate‐exposed patients may have rendered them collectively less fit for chemotherapy, which would exert a mitigating impact on any bias from PD‐L1 expression. Tobacco abuse constitutes another unmeasured confounder, as smoking can both cause hypertriglyceridemia and increase ICI response. However, the adverse prognostic impact of tobacco abuse and hypertriglyceridemia on OS would moderate this bias.

Third, only a small portion of our cohort received fibrate, introducing the potential for bias from imbalanced covariates. This limited utilization of fibrate therapy likely reflects the aforementioned management paradigms for lipid disorders, and we attempted to mitigate this bias with multiple means of statistical adjustment.

A fourth limitation lies in the variation in fibrates studied between prior preclinical research and our analysis of clinical outcomes. The fibrate studied in the Japanese mouse models was bezafibrate, which was unavailable in the US during the study period. Instead, only gemfibrozil and fenofibrate have been approved for use in the US and were prescribed to our population of Veterans. Unique among fibrates, bezafibrate activates all three PPAR isoforms (α, γ, δ) roughly equally, and this difference in agents raises the question of generalizability from mouse models to our observations in humans. Importantly, however, the α isoform exhibits the strongest association with mitochondrial metabolism and is activated by all fibrate agents,^19^ suggesting a common target in PPARα that mediates mitochondrial metabolic reprogramming, T‐cell reinvigoration, and ultimately improved outcomes with ICI.^20^ Moreover, preclinical enhancement of anti‐PD‐1 effect has also been observed with fenofibrate.^11^

Fifth, the findings from our nested cohorts of Veterans may not be directly generalizable to the broader NSCLC population. However, in consisting primarily of Caucasian males over the age of 65 with multiple medical problems including stage IV disease with adenocarcinoma histology, our cohort mirrors the characteristics of the larger NSCLC population in the West.^21^

Finally, the retrospective design and broad geographic distribution of the study population prevent us from exploring the biological mechanisms by which fibrates modulate the efficacy of ICI in our cohort. We are therefore unable to compare correlates of immune function between fibrate‐exposed and ‐unexposed patients. It is also possible that the observed association between fibrates and OS is mediated by pathways other than immune response. Nevertheless, our findings are consistent with preclinical immunological evidence demonstrating improved outcomes with the addition of fibrates to ICI.

In conclusion, this analysis demonstrated that fibrates given concomitant to ICI in NSCLC are associated with improved OS. Our findings are concordant with preclinical evidence that PPAR agonists restore oxidative metabolism and tumoricidal efficacy in exhausted cytotoxic T‐lymphocytes. The hazard ratio observed with fibrates is clinically meaningful and in line with the intended efficacy for studies of novel combinations that are ongoing. Additional prospective study is warranted for this novel application.

## AUTHOR CONTRIBUTIONS

William A. Stokes contributed conceptualization, methodology, investigation, writing (original draft, review, editing). Madhusmita Behera contributed conceptualization, methodology, writing (review, editing). Renjian Jiang contributed data curation, methodology, formal analysis, data curation, visualization. David A. Gutman contributed data curation, software, formal analysis, supervision. Zhonglu Huang contributed methodology, formal analysis, data curation, visualization. Abigail Burns contributed project administration, resources. Nikhil T. Sebastian contributed conceptualization, methodology, investigation, writing (review, editing). Vidula Sukhatme contributed conceptualization, funding acquisition. Michael C. Lowe contributed conceptualization, methodology, investigation. Suresh S. Ramalingam contributed conceptualization, methodology, investigation, writing (review, editing). Vikas P. Sukhatme contributed conceptualization, funding acquisition, methodology, writing (review, editing). Drew Moghanaki contributed conceptualization, funding acquisition, methodology, investigation, writing (original draft, review, editing).

## CONFLICT OF INTEREST

William A. Stokes has no disclosures. Madhusmita Behera has no disclosures. Renjian Jiang has no disclosures. David A. Gutman has no disclosures. Zhonglu Huang has no disclosures. Abigail Burns has no disclosures. Nikhil T. Sebastian has no disclosures. Vidula Sukhatme has no disclosures. Michael C. Lowe has no disclosures. Suresh S. Ramalingam has received grant funding and/or other support (for consultancy) from Amgen, AstraZeneca, Bristol‐Myers Squibb, Merck, Takeda, Tesaro, Advaxis, AbbVie, and Genentech/Roche. Vikas P. Sukhatme is on the SAB of BERG and HiFiBio Therapeutics, and an equity holder in Aggamin Pharmaceuticals and Victa Biotherapeutics. Drew Moghanaki has received travel support and speaking honoraria from Varian Medical Systems.

## ETHICAL APPROVAL STATEMENT

This study was conducted with the approval of the institutional review board of Emory University prior to data acquisition and analysis, and it conforms to the standards specified in the Declaration of Helsinki.

## INFORMED CONSENT

A waiver of informed consent was obtained for this retrospective cohort analysis of “on‐the‐shelf” data.

## Supporting information


Table S1‐S4

## Data Availability

The data underlying this article were provided by the Veterans Health Administration with permission. Data will be shared on request to the corresponding author with permission of the Department of Veterans Affairs.
