# Supplementary material for: Impact of concomitant fibrates on immunotherapy outcomes for advanced non‐small cell lung cancer
Source: Cancer Med. 2022 May 24;12(1):358–67. doi: 10.1002/cam4.4847 (PMC9844615; doi:10.1002/cam4.4847)
Supplement: Supplementary file 1 — Table S1‐S4 [file CAM4-12-358-s001.docx]

**Supplemental Table 1**. Covariate balance in propensity-matched cohort treated with immune checkpoint inhibitors

|  | | | Fibrate | | |  | | |
| --- | --- | --- | --- | --- | --- | --- | --- | --- |
|  | | | __________________________ | | |  | | |
| Covariate | Categories | No  N=298 | | Yes  N=298 | Parametric *P*-value* | | Standardized Difference |  |
| Chemotherapy | none | 48 (16.1) | | 55 (18.5) | 0.672 | | 0.062 |  |
|  | before ICI | 151 (50.7) | | 138 (46.3) |  |  | 0.087 |  |
|  | during ICI | 95 (31.9) | | 99 (33.2) |  |  | 0.029 |  |
|  | after ICI | 4 (1.3) | | 6 (2.0) |  |  | 0.052 |  |
| Age | ≤65 | 42 (14.1) | | 49 (16.4) | 0.567 | | 0.065 |  |
|  | 66-70 | 100 (33.6) | | 97 (32.6) |  |  | 0.021 |  |
|  | 71-75 | 83 (27.9) | | 91 (30.5) |  |  | 0.059 |  |
|  | >75 | 73 (24.5) | | 61 (20.5) |  |  | 0.097 |  |
| Race | other | 5 (1.7) | | 4 (1.3) | 0.860 | | 0.028 |  |
|  | black | 47 (15.8) | | 41 (13.8) |  |  | 0.057 |  |
|  | unknown | 10 (3.4) | | 12 (4.0) |  |  | 0.036 |  |
|  | white | 236 (79.2) | | 241 (80.9) |  |  | 0.042 |  |
| Geography | rural | 106 (35.6) | | 110 (36.9) | 0.733 | | 0.028 |  |
|  | urban | 192 (64.4) | | 188 (63.1) |  |  | 0.028 |  |
| Employment | employed | 54 (18.1) | | 49 (16.4) | 0.943 | | 0.044 |  |
|  | not employed | 125 (41.9) | | 131 (44.0) |  |  | 0.041 |  |
|  | retired | 112 (37.6) | | 111 (37.3) |  |  | 0.007 |  |
|  | unknown | 7 (2.4) | | 7 (2.4) |  |  | 0.000 |  |
| Marital Status | not married | 139 (46.6) | | 145 (48.7) | 0.623 | | 0.040 |  |
|  | married | 159 (53.4) | | 153 (51.3) |  |  | 0.040 |  |
| Gender | female | 0 (0.0) | | 2 (0.7) | 0.157 | | 0.116 |  |
|  | male | 298 (100.0) | | 296 (99.3) |  |  | 0.116 |  |
| Elixhauser Index | 0-4 | 43 (14.4) | | 47 (15.8) | 0.534 | | 0.037 |  |
|  | 5-6 | 55 (18.5) | | 67 (22.5) |  |  | 0.100 |  |
|  | 7-9 | 85 (28.5) | | 82 (27.5) |  |  | 0.022 |  |
|  | 10+ | 115 (38.6) | | 102 (34.2) |  |  | 0.091 |  |
| Histology | others | 49 (16.4) | | 51 (17.1) | 0.871 | | 0.018 |  |
|  | adenocarcinoma | 148 (49.7) | | 152 (51.0) |  |  | 0.027 |  |
|  | squamous cell carcinoma | 101 (33.9) | | 95 (31.9) |  |  | 0.043 |  |
| Stage at Diagnosis | I | 37 (12.4) | | 44 (14.8) | 0.841 | | 0.069 |  |
|  | II | 13 (4.4) | | 17 (5.7) |  |  | 0.061 |  |
|  | III | 84 (28.2) | | 81 (27.2) |  |  | 0.023 |  |
|  | IV | 116 (38.9) | | 109 (36.6) |  |  | 0.048 |  |
|  | unknown | 48 (16.1) | | 47 (15.8) |  |  | 0.009 |  |
| Year of Diagnosis | 2010-2015 | 114 (38.3) | | 124 (41.6) | 0.403 | | 0.069 |  |
|  | 2016-2018 | 184 (61.7) | | 174 (58.4) |  |  | 0.069 |  |
| Month from Diagnosis to ICI | 0-4 | 89 (29.9) | | 92 (30.9) | 0.925 | | 0.022 |  |
|  | 5-10 | 68 (22.8) | | 63 (21.1) |  |  | 0.041 |  |
|  | 11-19 | 65 (21.8) | | 62 (20.8) |  |  | 0.025 |  |
|  | ≥20 | 76 (25.5) | | 81 (27.2) |  |  | 0.038 |  |
| *  The parametric p value is calculated by Chi-Square test for categorical covariates.  Abbreviations: ICI, immune checkpoint inhibitor | | | | | | | | |

**Supplemental Table 2**. Descriptive statistics of unmatched cohort receiving docetaxel, stratified by fibrate exposure (χ^2^)

|  |  | Fibrate | | | |  |
| --- | --- | --- | --- | --- | --- | --- |
| Variable | Categories | No  *N*=887 | | Yes  *N*=81 | |  |
|  |  | *N* | % | *N* | % | *p* |
| Age | ≤65  66-70  71-75  >75 | 450  203  122  112 | 50.7  22.9  13.8  12.6 | 33  25  11  12 | 40.7  30.9  13.6  14.8 | 0.287 |
| Race | white  black  other  unknown | 636  204  15  32 | 71.7  23.0  1.7  3.6 | 65  9  1  6 | 80.2  11.1  1.2  7.4 | 0.040 |
| Gender | male  female | 867  20 | 97.7  2.3 | 78  3 | 96.3  3.7 | 0.412 |
| Geography | urban  rural | 591  296 | 66.6  33.4 | 49  32 | 60.5  39.5 | 0.264 |
| Employment | employed  not employed  retired  unknown | 179  408  288  12 | 10.2  46.0  32.5  1.4 | 14  34  33  0 | 17.3  42.0  40.7  0.0 | 0.361 |
| Marital Status | married  not married  unknown | 411  478  0 | 46.3  33.7  0.0 | 43  38  0 | 53.1  46.9  0.0 | 0.244 |
| Elixhauser Comorbidity Index | 0-3  4-5  6-7  >7 | 243  273  176  195 | 27.4  30.8  19.8  22.0 | 12  21  21  27 | 14.8  25.9  25.9  33.3 | 0.014 |
| Histology | squamous cell carcinoma  adenocarcinoma  other | 266  455  166 | 30.0  51.3  18.7 | 28  35  18 | 34.6  43.2  22.2 | 0.377 |
| Stage at Diagnosis | 0  I  II  III  IV  unknown | 0  0  0  0  887  0 | 0.0  0.0  0.0  0.0  100.0  0.0 | 0  0  0  0  81  0 | 0.0  0.0  0.0  0.0  100.0  0.0 | 1.000 |
| Year of Diagnosis | 2010-2015  2016-2018 | 844  43 | 95.2  4.8 | 77  4 | 95.1  4.9 | 0.971 |
| Months from Diagnosis to Docetaxel | 0-6  7-12  13-24  >24 | 837  28  17  5 | 94.4  3.2  1.9  0.6 | 78  3  0  0 | 96.3  3.7  0.0  0.0 | 0.550 |

**Supplemental Table 3**. Cox regression analysis of overall survival for unmatched patients treated with docetaxel

| Variable | Categories |  | UVA | | | MVA | | | |
| --- | --- | --- | --- | --- | --- | --- | --- | --- | --- |
|  |  | *N* | HR | 95%CI | *p* | HR | 95%CI | *p* | *pT3* |
| Fibrate | no  yes | 81  887 | -  1.02 | -  0.81-1.28 | -  0.855 | -  0.99 | -  0.79-1.25 | -  0.962 | 0.962 |
| Age | ≤65  66-70  71-75  >75 | 483  228  133  124 | 0.94  0.85  0.75  - | 0.77-1.15  0.68-1.06  0.58-0.96  - | 0.543  0.146  0.021  - | 0.99  0.86  0.78  - | 0.81-1.21  0.69-1.08  0.61-1.01  - | 0.903  0.195  0.055  - | 0.061 |
| Race | white  black  other  unknown | 701  213  16  38 | -  0.92  1.39  0.95 | -  0.79-1.07  0.85-2.28  0.68-1.31 | -  0.193  0.290  0.738 |  |  |  |  |
| Gender | male  female | 945  23 | -  0.67 | -  0.43-1.02 | -  0.060 |  |  |  |  |
| Geography | urban  rural | 640  328 | -  1.03 | -  0.90-1.17 | -  0.694 |  |  |  |  |
| Employment | employed  not employed  retired  unknown | 193  442  321  12 | -  0.97  0.97  0.57 | -  0.82-1.15  0.81-1.16  0.31-1.04 | -  0.700  0.740  0.067 |  |  |  |  |
| Marital Status | married  not married | 454  514 | -  1.01 | -  0.89-1.15 | -  0.896 |  |  |  |  |
| Elixhauser Comorbidity Index | 0-3  4-5  6-7  >7 | 294  197  222  255 | -  1.18  1.24  1.13 | -  1.00-1.40  1.03-1.50  0.94-1.35 | -  0.057  0.025  0.187 |  |  |  |  |
| Histology | squamous cell carcinoma  adenocarcinoma  other | 294  490  184 | -  0.76  0.95 | -  0.65-0.87  0.79-1.14 | -  <0.001  0.595 | -  0.76  0.95 | -  0.66-0.88  0.79-1.15 | -  <0.001  0.620 | <0.001 |
| Year of Diagnosis | 2010-2015  2016-2018 | 921  47 | 0.66  - | 0.49-0.89  - | 0.007 | 0.67  - | 0.49-0.91  - | 0.010  - | 0.010 |
| Months from Diagnosis to Docetaxel | 0-6  7-12  13-24  >24 | 915  31  17  5 | 1.84  1.83  2.65  - | 0.69-4.90  0.65-5.19  0.89-7.87  - | 0.226  0.254  0.080  - |  |  |  |  |
| Abbreviations: HR, hazard ratio; MVA, multivariable analysis; pT3, type-3 *p-*value; UVA, univariable analysis | | | | | | | | | |

**Supplemental Table 4**. Covariate balance in propensity-matched cohort treated with docetaxel

|  | | | Fibrate | | |  | | |
| --- | --- | --- | --- | --- | --- | --- | --- | --- |
|  | | | __________________________ | | |  | | |
| Covariate | Categories | No  N=79 | | Yes  N=79 | Parametric *P*-value* | | Standardized Difference |  |
| Age | ≥65 | 33 (41.8) | | 32 (40.5) | 0.947 | | 0.026 |  |
|  | 66-70 | 21 (26.6) | | 24 (30.4) |  |  | 0.084 |  |
|  | 71-75 | 11 (13.9) | | 11 (13.9) |  |  | 0.000 |  |
|  | >76 | 14 (17.7) | | 12 (15.2) |  |  | 0.068 |  |
| Race | black | 8 (10.1) | | 9 (11.4) | 0.748 | | 0.041 |  |
|  | unknown | 5 (6.3) | | 6 (7.6) |  |  | 0.050 |  |
|  | other | 0 (0.0) | | 1 (1.3) |  |  | 0.160 |  |
|  | white | 66 (83.5) | | 63 (79.7) |  |  | 0.098 |  |
| Gender | female | 1 (1.3) | | 1 (1.3) | 1.000 | | 0.000 |  |
|  | male | 78 (98.7) | | 78 (98.7) |  |  | 0.000 |  |
| Geography | rural | 31 (39.2) | | 32 (40.5) | 0.871 | | 0.026 |  |
|  | urban | 48 (60.8) | | 47 (59.5) |  |  | 0.026 |  |
| Employment Status | employed | 10 (12.7) | | 14 (17.7) | 0.666 | | 0.141 |  |
|  | not employed | 33 (41.8) | | 32 (40.5) |  |  | 0.026 |  |
|  | retired | 36 (45.6) | | 33 (41.8) |  |  | 0.077 |  |
| Marital Status | not married | 38 (48.1) | | 38 (48.1) | 1.000 | | 0.000 |  |
|  | married | 41 (51.9) | | 41 (51.9) |  |  | 0.000 |  |
| Elixhauser Index | 0-3 | 14 (17.7) | | 12 (15.2) | 0.726 | | 0.068 |  |
|  | 4-5 | 17 (21.5) | | 21 (26.6) |  |  | 0.119 |  |
|  | 6-7 | 25 (31.7) | | 20 (25.3) |  |  | 0.141 |  |
|  | 8+ | 23 (29.1) | | 26 (32.9) |  |  | 0.082 |  |
| Histology | adenocarcinoma | 35 (44.3) | | 35 (44.3) | 0.910 | | 0.000 |  |
|  | others | 19 (24.1) | | 17 (21.5) |  |  | 0.060 |  |
|  | squamous cell carcinoma | 25 (31.6) | | 27 (34.2) |  |  | 0.054 |  |
| Year of Diagnosis | 2010-2015 | 77 (97.5) | | 75 (94.9) | 0.405 | | 0.133 |  |
|  | 2016-2018 | 2 (2.5) | | 4 (5.1) |  |  | 0.133 |  |
| Months from Diagnosis to Docetaxel | 0-6 | 75 (94.9) | | 76 (96.2) | 0.699 | | 0.062 |  |
|  | 7-12 | 4 (5.1) | | 3 (3.8) |  |  | 0.062 |  |
|  | | | | | | | | |
| *  The parametric p value is calculated by Chi-square test for categorical covariates. | | | | | | | | |
